# Sex hormone binding globulin (SHBG) serum levels and insulin resistance in men on chronic hemodialysis

**DOI:** 10.1186/s13098-024-01406-9

**Published:** 2024-07-16

**Authors:** Evdokia Nikolaou, Maria Tziastoudi, Sofia G. Gougoura, Georgios Filippidis, Periklis Dousdampanis, Alexandra Bargiota, Peter Rene Mertens, Theodoros Eleftheriadis, Georgios M. Hadjigeorgiou, Georgios N. Koukoulis, Ioannis Stefanidis

**Affiliations:** 1 Department of Nephrology, University of Thessaly School of Medicine, Mezourlo Hill, Larissa, 41110 Greece; 2 Department of Endocrinology, University of Thessaly School of Medicine, Larissa, Greece; 3 Department of Nephrology, Saint Andrews State General Hospital, Patras, 26221 Greece; 4 Department of Nephrology, Hypertension, Diabetes and Endocrinology, School of Medicine, University of Magdeburg, Magdeburg, Germany; 5 Department of Neurology, Medical School, University of Cyprus, Nicosia, 2408 Cyprus

**Keywords:** Estradiol, Hemodialysis, Homeostasis model, Insulin resistance, Male hypogonadism, Testosterone, Uremia

## Abstract

**Background:**

In males with end stage renal disease biochemical hypogonadism is a frequent finding. Testosterone and sex hormone binding globulin (SHBG) have been associated with insulin resistance, a well-known condition in uremia. The aim of the present study was to investigate in males on chronic hemodialysis the relationship of testosterone and SHBG serum levels with insulin resistance.

**Methods:**

In a cross-sectional study we enrolled men treated with chronic hemodialysis who did not suffer from an acute illness or other endocrinopathy, as well as primary hypogonadism, and were not hospitalised. Diabetes mellitus, diabetic nephropathy or previous transplantation were not exclusion criteria. As controls we used a community-based group of healthy males matched for age and Body Mass Index (BMI). We assessed the BMI (kg/m^2^) from body weight and height, the body fat content (%) by bioelectrical impedance and serum testosterone (ng/ml), SHBG (nmol/L) and estradiol (pg/ml) by standard methods. Testosterone < 3.25 ng/ml defined biochemical hypogonadism. In non-diabetic males, we calculated the homeostasis model assessment index (HOMA-R), an estimate of insulin resistance, from serum fasting insulin and glucose.

**Results:**

27 men (age 54.4 ± 19 years) on chronic hemodialysis (treatment duration 29.1 ± 14.4 months) and 51 healthy men (age 47.1 ± 9.6 years) were included. In men on hemodialysis vs. healthy men there were increased serum levels of SHBG (40.9 ± 26.9 vs. 27.6 ± 11.9 nmol/L; *p* = 0.031) and a significantly enhanced frequency of biochemical hypogonadism (22.2 vs. 3.9%; *p* = 0.011). In cases without diabetes (*n* = 22) a significant correlation was observed between the HOMA-R (*r* = -0.586, *p* = 0.004) and the fasting insulin levels (*r* = -0.650, *p* = 0.001) on the one hand and the serum SHBG levels on the other.

**Conclusions:**

Our findings confirm enhanced prevalence of biochemical hypogonadism in males on chronic hemodialysis. In non-diabetic cases the serum levels of SHBG correlated with serum insulin and insulin resistance.

## Background

Insulin resistance is the impaired ability of plasma insulin to promote tissue glucose disposal adequately and is accompanied frequently by hyperinsulinemia and glucose intolerance. In chronic kidney disease and uremia insulin resistance is very common [1, 2]. Hemodialysis treatment, by significantly improving uremia, ameliorates insulin resistance [3]. Previous clinical and experimental studies suggest that in kidney disease the main site of insulin resistance is the skeletal muscle, where a defect of the post-receptor pathway is involved [4, 5]. However, the exact pathogenic mechanism of insulin resistance in kidney disease remains still unresolved.

In men with severe chronic kidney disease there is frequently a biochemical primary and a concurrent secondary hypogonadism [6, 7]. Low serum testosterone in men has been associated with the metabolic syndrome, diabetes mellitus and insulin resistance [8–10]. In the same context, sex hormone binding globulin (SHBG), a plasma transporter of sex steroids, is also associated with the metabolic syndrome and with insulin resistance [11, 12]. SHBG plasma concentration, independently from sex steroids, is inversely correlated with glycosylated hemoglobin in both men and women without diabetes mellitus. In women with polycystic ovarian syndrome, a condition associated with insulin resistance and an increased risk of type 2 diabetes mellitus, SHBG concentrations are decreased [13]. Furthermore, hypogonadism is a recognised factor predisposing to increased mortality and cardiovascular morbidity both in males undergoing chronic hemodialysis and in males of the general population [14].

SHBG is a 90-kD glycoprotein composed of two 373-amino-acid subunits, each with a single steroid binding site. SHBG is synthesized mainly in hepatocytes, and its clearance from the intravascular compartment is dependent on glycosylation, which prolongs half life. The kidneys, however, are not involved either in synthesis or in catabolism and clearance of the molecule. Traditionally, it has been considered to function merely as a sex steroid transporter, controlling circulating free sex steroids in blood. However, today certain findings suggest that SHBG has additional functions [15]. The existence of a plasma membrane receptor binding unliganded SHBG was previously demonstrated. Liganded SHBG does not bind to the SHBG-receptor on the cellular plasma membrane, suggesting that there are SHBG cellular effects, which are independent of sex steroids. In addition, binding of a sex steroid on the SHBG bound on its receptor leads to an activation of a G-protein-linked second-messenger system. The resulting cellular reactions depend on the sex steroid ligand. The presence of an SHBG receptor in the plasma membrane and of a second messenger system provides a probable explanation for the non-genomic actions of sex hormones [16].

The aim of the present cross-sectional study in men with end stage renal disease on chronic hemodialysis was to address the hypothesis that testosterone and SHBG serum levels may relate to insulin resistance, a very common condition in uremia and investigate a probable relationship of biochemical hypogonadism with insulin resistance in this group.

## Methods

### Subjects

In this cross-sectional study the inclusion criteria were male patients with end-stage renal disease treated with chronic hemodialysis in the Renal Unit at the University Hospital of Larissa. All were enrolled if they were on treatment with chronic hemodialysis treatment for more than three months, did not suffer from an acute illness or other endocrinopathy, as well as primary hypogonadism, and were not hospitalised. Patients were excluded if they were receiving hemodialysis treatment transiently, i.e. for acute renal failure, and if they were unable to provide informed consent. None of the patients was previously transplanted. Tre presence of diabetes mellitus/diabetic nephropathy was not exclusion criteria.

Relevant clinical and laboratory data as well as 5-years mortality data were recorded from the medical records kept in the renal unit. If this was not possible mortality data were acquired from the national end stage renal disease registry. A community based control group of healthy males matched for age (± 5 years) and Body Mass Index (BMI; ±2 kg/m^2^) was used.

The purpose and the procedure of the study were clearly explained to the participants, who then all provided informed consent. Before initiation, the study presented here was approved by the Ethics Review Board of the University Hospital of Larissa.

### Clinical and laboratory data

We assessed Body Mass Index (BMI; kg/m^2^) according to the Quetelet’s formula from body weight (W; kg) and height (H; m) [17]: $BMI = {W \over {{H^2}}}$. For body fat content (%) we applied a bioelectrical impedance analysis by means of a commercially available bio-impedance analyser (Omron HBF 302 Body Fat Analyser, Omron Healthcare Inc., Vernon Hills, IL, USA) with the participant in standing position. Waist circumference (cm) was measured according to the World Health Organisation (WHO) *step*wise approach to *s*urveillance (STEPS) [18] using a plastic non-stretchable tailor’s measuring tape, at the approximate midpoint between the lower margin of the last palpable rib margin and the top of the iliac crest to the nearest centimetre, as previously described. In hemodialysis patients waist circumference (cm), body weight (kg) and body fat content (%) were assessed after the end of the hemodialysis session [19].

The protein catabolic rate (PCR) was calculated from the pre-dialysis BUN concentration (C_0_; mg/dl) and the Kt/V [20], based on a two-BUN measurement, single-pool, variable-volume model by the following formula, for patients dialysed thrice weekly and especially for a C_0_ measured after the long dialysis-free interval at the beginning of the week:

$$PCR = \frac{{{C_0}}}{{36.3 + 5.48 \cdot Kt/V + \frac{{53.5}}{{Kt/V}}}} + 0.168$$

Hemodialysis adequacy was assessed by the urea clearance ratio (Kt/V), which was calculated from the urea reduction ratio (R), the ultrafiltration volume (UF) and the post-dialysis body weight (W) *via* the second generation natural logarithmic formula, also based on the single pool urea kinetic model, namely:

$$\begin{aligned}& Kt/V = \\ & - \ln \left( {R - 0.008 \cdot t} \right) + \left( {4 - 3.5 \cdot R} \right) \cdot \frac{{UF}}{W} \\ \end{aligned}$$

Blood samples were always collected after an overnight fast in the morning (8–10 am) and in hemodialysis patients, especially, before commencement of the middle week (Wednesday or Thursday) hemodialysis session. All parameters in serum samples were measured with commercially available assays. Total testosterone (ng/ml) and total estradiol serum levels (pg/ml) were assessed by a coat-a-count solid phase radioimmunoassay (RIA; Diagnostic System Laboratories Inc., Webster, TX, USA). The assay sensitivity for serum testosterone was 0.08 ng/ml and for serum estradiol 0.6 pg/ml. The interassay coefficient of variation (CV) was 8% for serum testosterone and 4% for serum oestradiol measurements. Serum levels of sex hormone binding globulin (SHBG; nmol/L) were measured using an immunoradiometric assay (IRMA; Radim SpA, Roma, Italy). The assay sensitivity was 2.5 nmol/L and the interassay CV 5%. Free androgen index (FAI) was the ratio of serum total testosterone (nmol/L) to serum SHBG (nmol/L). According to previous reports, biochemical hypogonadism was defined either as serum total testosterone ≤ 3.25 ng/ml or as FAI ≤ 0.153 [21].

In non-diabetic men serum glucose levels were determined by means of the Glucoanalyzer II (Beckmann, Munich, Germany) and the serum insulin concentrations by an IRMA (Immunotech, Beckman Counter Company, Marseille, France). From the serum fasting levels of glucose (Glucose; mg/dl) and insulin (Insulin; µIU/ml) the homeostasis model assessment ratio (HOMA-R) was calculated according to the following formula: $HOMA - R = {{Glu\cos e \cdot Insulin} \over {405}}$. The HOMA-R (mmol·µIU/ml) was previously validated in 888 healthy Caucasian subjects and the values in the top quintile of the distribution (> 2.77 mmol·µIU/ml) were defined as insulin resistant state, while lower values were considered to represent an insulin sensitive state.

### Statistical analysis

Data were expressed as mean values ($\bar {\rm X}$) with standard deviation (SD). The normality of continuous variables was tested by the Kolmogorov-Smirnov test. Variables not following normal distribution were logarithmically transformed for implementation in linear regression. Pair-wise comparisons of continuous variables were performed with the t-test or the Mann-Whitney U test for unpaired data, as appropriate. For comparison of categorical variables among the two groups we used the χ^2^ test. Interdependence of the different continuous variables was checked by means of a Pearson or a Spearman correlation analysis and a linear regression analysis, as appropriate. Based on the results of the univariate analysis and respective scatter-plots we set up a multiple linear regression model. In the model, we included HOMA-R (mmol·µIU/ml) as the dependent variable on the one hand and age (years), testosterone (ng/ml) as well as all parameters that significantly correlated with HOMA-R (mmol·µIU/ml) as predictor variables on the other.

Overall, statistical analyses were carried out using the Statistic Package for Social Sciences 13.0 (SPSS^®^ 13.0) for Windows (SPSS Inc., Chicago, IL, USA). P-values < 0.05 were considered statistically significant.

## Results

**Fig. 1:**
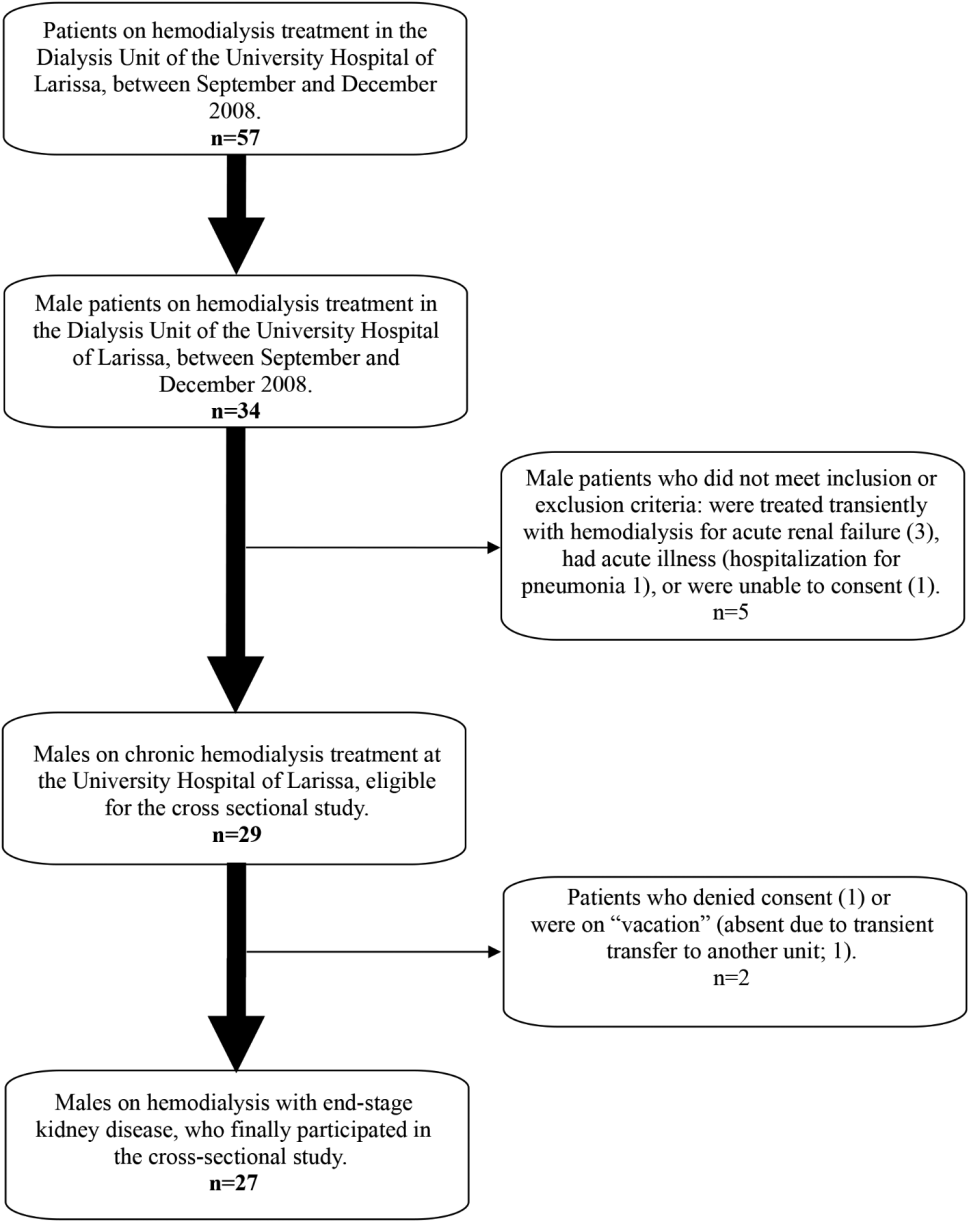
Flowchart of participants in the cross-sectional study at the Dialysis Unit of the University Hospital of Larissa

The flowchart of the study participation among hemodialysis patients is shown in Fig. 1. We enrolled 27 men (age 54.4 ± 19 years) on chronic hemodialysis (treatment duration 29.1 ± 14.4 months) and 51 healthy men (age 47.1 ± 9.6 years). On Table 1 primary diseases of men on chronic hemodialysis, enrolled in the study, is shown.

**Table 1:** Primary disease of men with end-stage renal disease treated with chronic hemodialysis (males on HD; *n* = 27) in the study

		Males on HD		
Primary disease	total	TT ≤ 3.25ng/ml	TT > 3.25ng/ml	*p*-value1
Vascular renal disease	5 (18.5)	2 (20)	3 (17.6)	
Diabetic nephropathy	4 (14.8)	3 (30)	1 (5.9)	
Chronic glomerulonephritis	5 (18.5)	3 (30)	2 (11.8)	
Systematic disease	4 (14.8)	0 (0)	4 (23.5)	
Interstitial nephritis/Obstructive uropathy	2 (7.4)	0 (0)	2 (11.8)	0.291
Polycystic kidney disease	2 (7.4)	1 (10)	1 (5.9)	
Alport’s syndrome	2 (7.4)	0 (0)	2 (11.8)	
Other causes/Unknown etiology	3 (11.1)	1 (10)	2 (11.8)	
Sum	27 (100)	10 (100)	17 (100)	

1 p-values from the χ2 test. Variables are given as number (n) and frequency as percentage in brackets (%). HD = hemodialysis, TT = total testosterone

Prevalence of biochemical hypogonadism (testosterone < 3.25 ng/ml) was increased in men on hemodialysis vs. healthy men (22.2 vs. 3.9%; *p* = 0.011). Furthermore, in men on hemodialysis there were significantly increased serum levels of SHBG (nmol/L; *p* = 0.031) along with increased free testosterone index values and significantly reduced testosterone (ng/ml) and estradiol levels (pg/ml). All these findings are presented in detail on Table 2.

**Table 2:** Clinical and laboratory profiles of men with end-stage renal disease that undergo chronic hemodialysis treatment (*n* = 27) and of healthy men (*n* = 51) enrolled in the cross-sectional study

	Healthy males	Males on HD	*p*-value	Males on HD	Males on HD	*p*-value
**Variables (units)**	(*n* = 51)	(*n* = 27)		TT ≤ 3.25ng/ml (*n* = 10)	TT > 3.25ng/ml (*n* = 17)	
*Age (years)*	47.1 ± 9.6	54.4 ± 19.0	0.089	58.5 ± 15.0	52.1 ± 21.5	0.604
*BMI (kg/m2)*	26.1 ± 3.0	24.4 ± 4.6	0.016	25.2 ± 3.1	24.4 ± 4.6	0.204
*Fat content (%)*	21.1 ± 5.4	24.5 ± 9.0	0.065	25.2 ± 3.1	24.0 ± 5.4	0.367
*Mean time on HD (months)*	n.a.	29.1 ± 14.4	n.a.	27.5 ± 6.2	29.0 ± 15.0	0.264
*KT/V*	-	1.21 ± 0.23	-	1.31 ± 0.25	1.17 ± 0.22	0.231
*PCR (g/kg/d)*	-	1.07 ± 0.25	-	1.15 ± 0.25	1.03 ± 0.25	0.294
*Albumin (mg/dl)*	-	4.06 ± 0.47	-	3.93 ± 0.68	4.13 ± 0.30	0.786
*C-Reactive Protein (CRP; mg/dl)*	-	0.74 ± 0.95	-	0.82 ± 0.87	0.70 ± 1.01	0.339
**Endocrinological variables (units)**	**(n = 51)**	**(n = 27)**		**TT ≤ 3.25ng/ml (n = 10)**	**TT > 3.25ng/ml (n = 17)**	
*SHBG (nmol/L)*	27.6 ± 11.9	40.9 ± 26.9	0.031	37.7 ± 36.6	42.8 ± 20.3	0.170
*Total Testosterone (TT; ng/ml)*	6.2 ± 2.0	3.6 ± 1.5	< 0.001	2.3 ± 1.1	4.4 ± 1.0	< 0.001
*Total Estradiol (pg/ml)*	25.2 ± 6.0	15.9 ± 6.1	< 0.001	14.3 ± 6.0	16.9 ± 6.1	0.414
*Free androgen index (FAI)*	0.85 ± 0.30	0.50 ± 0.45	< 0.001	0.47 ± 0.47	0.51 ± 0.45	0.473
*Hypogonadism (TT ≤ 3.25 ng/ml)*	3 (5.9)	10 (37.0)	< 0.001	10 (100)	0 (0)	n.a.
*Hypogonadism (FAI < 0.153)*	0 (0)	4 (14.8)	0.005	3 (75)	1 (25)	0.128
	**(n = 51)**	**no DM (n = 22)**		**(n = 6)**	**(n = 16)**	
*Insulin (µIU/L)*	6.56 ± 3.91	8.19 ± 5.37	0.279	9.80 ± 6.25	7.59 ± 5.10	0.641
*Glucose (mg/dl)*	88 ± 11	79 ± 10	< 0.001	86 ± 13	76 ± 8	0.059
*HOMA-R (mmol∙µIU/ml)*	1.45 ± 0.91	1.61 ± 1.14	0.727	2.17 ± 1.58	1.41 ± 0.90	0.261
*Insulin resistance (> 2.77 mmol·µIU/ml)*	5 (9.8)	4 (18.2)	0.318	2 (33.3)	2 (12.5)	0.292

n.a.: not applicable. Values of continuous variables are given as mean and standard deviation (x ± SD). Categorical variables are given as number (n) and frequency as percentage in brackets (%). p-values from the Mann-Whitney U test for comparison of continuous variables and from the χ2 test for comparison of categorical variables

**Fig. 2:**
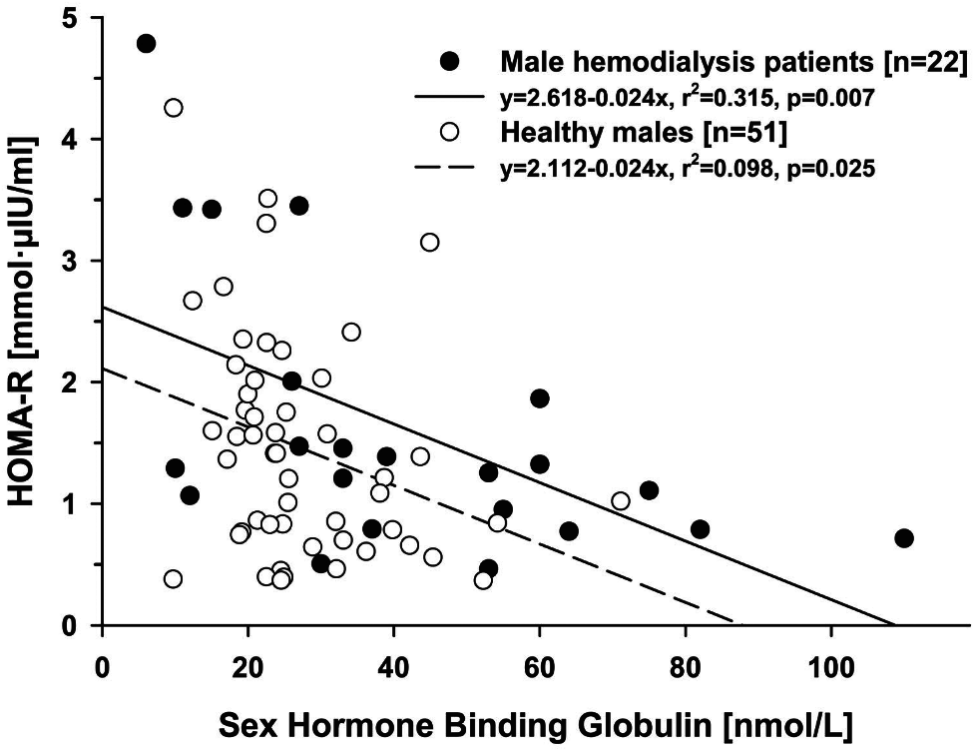
Association between the serum levels of Sex Hormone Binding Globulin (SHBG; nmol/ml) and the Homeostasis Model Assessment Ratio (HOMA-R; mmol·µIU/ml) in non-diabetix males on chronic hemodialysis treatment (n = 22) and in healthy men (n = 51)

In cases without diabetes mellitus (n = 22) a significant negative correlation was observed between insulin resistance expressed as the HOMA-R (mmol·µIU/ml; *r* = -0.586, *p* = 0.004) and the fasting insulin levels (µIU/L; *r* =-0.650, *p* < 0.001) on the one hand and the serum SHBG levels (nmol/L) on the other. In healthy males (n = 51) correlation analysis showed similar results (Table 3). The significant relationship between serum levels of SHBG (nmol/ml) and the Homeostasis Model Assessment Ratio (HOMA-R; mmol·µIU/ml) both in non-diabetic males on chronic hemodialysis treatment (n = 22) and in healthy men (n = 51) is presented graphically in Fig. 2. In addition, in non-diabetic hemodialysis patients (n = 22) there was significant correlation between the HOMA-R (mmol·µIU/ml) on the one hand and the BMI (kg/m^2^), the body fat content (%), the hemodialysis treatment duration (months) and the SHBG (nmol/L) on the other (Table 3).

**Table 3:** Correlation coefficients that describe the relationship between anthropometric, metabolic and hormonal parameters in non-diabetic men with end-stage renal disease on chronic hemodialysis treatment (n = 22) and in healthy men (n = 51)

	Correlation with1								
Variables (units)	Fasting insulin (µIU/L)		HOMA-*R* (mmol∙µIU/ml)			Fasting insulin (µIU/L)		HOMA-*R* (mmol∙µIU/ml)	
	in non-diabetic HD males (n = 22)					in healthy males (n = 51)			
	**r**	**p**	**r**	**p**		**r**	**p**	**r**	**p**
**Age (Years)**	0.013	0.955	0.106	0.639		0.126#	0.376#	0.132#	0.355#
**Time on dialysis (Months)**	0.373	0.087	0.437	0.042		-	-	-	-
**BMI (kg/m2)**	0.764	< 0.001	0.717	< 0.001		0.260	0.065	0.272	0.054
**Body fat content (%)**	0.614	0.002	0.581	0.005		0.278	0.048	0.279	0.047
**Waist circumference (cm)**	0.816	< 0.001	0.741	< 0.001		0.189	0.184	0.213	0.134
**SHBG (nmol/L)**	-0.588	0.004	-0.561	0.007		-0.376#	0.007#	-0.358#	< 0.001#
**Total Testosterone (ng/ml)**	-0.178	0.428	-0.281	0.205		-0.275	0.051	-0.267	0.058
**Free androgen index (FAI)**	0.555	0.007	0.525	0.012		0.215	0.129	0.198	0.164
**Total Estradiol (pg/ml)**	0.146	0.518	0.101	0.653		0.006	0.965	0.028	0.848

1 Pearson’s correlation coefficients (r) and respective p-values in variables following normal distribution

# Spearman’s correlation coefficients (r) and respective p-values. Spearman’s correlation is applied in variables not following normal distribution

The significant relationship between the SHBG levels (nmol/L) and insulin resistance, i.e. the HOMA-R (mmol·µIU/ml), persisted in multiple regression analysis. Concretely, both BMI (kg/m^2^) and SHBG levels (nmol/L) statistically significantly predicted HOMA-R (mmol·µIU/ml), F(4, 18) = 9.565, *p* < 0.001, r^2^ = 0.692. Each independent variable, i.e. BMI and SHBG levels, added statistically significantly to the prediction of HOMA-R (*p* < 0.05). Detailed findings of the multiple linear regression analysis including SHBG (nmol/L), age (years), dialysis duration (months), BMI (kg/m^2^), fat content (%), waist circumference (cm) as predictor variables and HOMA-R (mmol·µIU/ml) as dependent variable are shown on Table 4. Further multivariate models of regression analysis dealing with the relationship of the SHBG levels (nmol/L) with HOMA-R (mmol·µIU/ml) are shown on Table 5.

**Table 4:** Multiple linear regression analysis model including the serum levels of sex hormone binding globulin (SHBG; nmol/ml) as a predictor variable and insulin resistance expressed as homeostasis model assessment ratio (HOMA-R; mmol·µIU/ml) as the dependent variable in male non-diabetic patients with end-stage renal disease on chronic hemodialysis treatment (n = 22, r^2^ = 0.721, *p* = 0.004) and in healthy men (n = 51, r^2^ = 0.176, *p* = 0.179)

Linear regression model	Male non-diabetic HD patients (n = 22)					Male healthy controls (n = 51)			
	B	SE	*p*				B	SE	*p*
Intercept	-0.064	1.718	0.971				0.295	1.624	0.857
SHBG (nmol/ml)	-0.022	0.009	0.033				-0.018	0.015	0.234
Testosterone (ng/ml)	0.168	0.185	0.380				-0.002	0.092	0.983
Age (years)	0.012	0.015	0.420				-0.011	0.020	0.583
Dialysis duration (months)	-0.001	0.017	0.942				n.a.	n.a.	n.a.
Body Mass Index (kg/m2)	0.116	0.108	0.300				0.035	0.061	0.567
Fat content (%)	0.010	0.038	0.806				0.040	0.036	0.285
Waist circumference (cm)	-0.004	0.039	0.916				0.005	0.016	0.761

n.a.: not applicable

* In healthy males Model 7 is identical with Model 4 because the variable Dialysis duration is not applicable in this group

**Table 5:** Multiple linear regression analysis models (1–11) in male non-diabetic patients with end-stage renal disease on chronic hemodialysis treatment (n = 22) and in healthy men (n = 51). In all models, insulin resistance expressed as homeostasis model assessment ratio (HOMA-R; mmol·µIU/ml) was included as the dependent variable and the serum levels of sex hormone binding globulin (SHBG; nmol/ml) as an ubiquitous predictor variable

Linear regression models		Male non-diabetic HD patients (n = 22)					Male healthy controls (n = 51)				
		B (SE)	*r* ^2^	*p*				B (SE)	*r* ^2^	*p*	
**1**	**SHBG**	-0.24 (0.008)	0.315	0.007				-0.24 (0.010)	0.098	0.025	
**2**	**1 + testosterone**	-0.025 (0.007)	0.432	0.003				-0.019 (0.014)	0.106	0.177	
**3**	**1 + Age**	-0.034 (0.008)	0.495	< 0.001				-0.26 (0.011)	0.117	0.017	
**4**	**1 + Dialysis duration**	-0.020 (0.008)	0.395	0.020				n.a.	n.a.	n.a.	
**5**	**1 + BMI**	-0.015 (0.006)	0.620	0.032				-0.22 (0.010)	0.152	0.041	
**6**	**1 + Fat content**	-0.023 (0.006)	0.627	0.001				-0.22 (0.010)	0.160	0.036	
**7**	**1 + WC**	-0.013 (0.007)	0.619	0.078				-0.22 (0.010)	0.127	0.039	
8*	**4 + BMI**	-0.014 (0.007)	0.642	0.055				n.a.	n.a.	n.a.	
**9**	**8 + Fat content**	-0.017 (0.007)	0.690	0.021				-0.21 (0.010)	0.169	0.044	
**10**	**9 + WC**	-0.018 (0.008)	0.691	0.033				-0.21 (0.010)	0.170	0.049	
**11**	**10 + Age**	-0.022 (0.009)	0.705	0.032				-0.019 (0.011)	0.176	0.108	

n.a.: not applicable

* In healthy males Model 8 is identical with Model 5 because the variable Dialysis duration is not applicable in this group

## Discussion

Our study examines whether testosterone and SHBG serum levels may relate to insulin resistance in men on chronic hemodialysis. Findings, in agreement with previous studies, showed an enhanced prevalence of biochemical hypogonadism with significantly decreased total testosterone levels and free androgen index (FAI) in men on chronic hemodialysis in comparison to healthy males [22]. Primary hypogonadism is predominant in end stage renal disease, most probably due to local accumulation of uremic toxins causing testicular dysfunction [23]. The pathogenic role of uremia is obvious from studies showing a rapid recovery of hypogonadism in males after receiving a kidney transplant [24]. This notion is strongly supported by findings of significantly decreased testosterone and enhanced LH and FSH levels, which parallel the progression of CKD, which failed to correct after human chorionic gonadotropin (hCG) administration both in uremic rats and humans [24].

In our study, serum SHBG levels were significantly higher in men on chronic hemodialysis than in healthy men. This finding is in agreement with previous studies, although evidence may be contradicting. Furthermore, neither kidney transplantation nor worsening kidney function in chronic kidney disease had been shown to influence SHBG levels. However, according to recent lines of evidence, there are also indications of potential connections between androgens and SHBG levels and kidney dysfunction in both men and women [25]. Men exhibiting lower SHBG levels face an increased likelihood of having a low estimated glomerular filtration rate (eGFR), indicating diminished kidney function [26].

Furthermore, the SHBG levels in our study negatively correlated with the measures of insulin resistance – HOMA-R and serum insulin – both in healthy men and in non-diabetic men on chronic hemodialysis. In contrast, the levels of total testosterone did not correlate with measures of insulin resistance. In the multiple regression-analysis independent predictors of insulin resistance were both SHBG levels and BMI. The relationship between SHBG levels and insulin resistance was independent from total testosterone and from the free androgen index (FAI). In addition, the enhanced levels of SHBG in hemodialysis patients were inversely related to HOMA-R supporting the hypothesis that SHBG is pathogenetically involved, ameliorating uremic insulin resistance.

In this context, SHBG seems to be independently associated with insulin resistance in our study. In both men and women without diabetes mellitus SHBG concentrations were previously shown to be, independently from sex steroids, inversely correlated with glycated hemoglobin.

In a post-hoc analysis of the Evaluation of Cinacalcet Therapy to Lower Cardiovascular Events (EVOLVE) randomized controlled trial serum levels of total testosterone, free testosterone, and SHBG were examined in relation to cardiovascular outcomes. Reduced serum levels of free testosterone and elevated levels of SHBG in men undergoing chronic hemodialysis were linked to an increased risk for mortality and cardiovascular morbidity [27].

In a recent meta-analysis of cohort studies low levels of both total and free testosterone were prognostic indicators for adverse clinical events in male patients with CKD [28]. More specifically, for every 1-standard deviation (SD) decrease in total testosterone, there was an independent increase in the risk of all-cause mortality by 27%, cardiovascular mortality by 100%, cardiovascular events by 20%, and infectious events by 41%. Additionally, a 1-SD decrease in free testosterone corresponded to a 66% increase in the risk of overall adverse events. Subgroup analysis demonstrated that the inverse association between total testosterone and the risk of all-cause death remained significant regardless of factors such as age, race, body mass index, diabetes, hypertension, C-reactive protein, creatinine, and sex hormone binding globulin [28]. Similarly in an additional recent meta-analysis low levels of endogenous testosterone could independently predict adverse clinical outcomes among male patients with CKD [29].

Chronic kidney disease (CKD) is an inflammatory condition, and inflammation exhibits a negative correlation with circulating testosterone levels, which tends to intensify in advanced CKD. It remains uncertain however whether the reduced testosterone levels are a cause or a consequence of inflammation in CKD. In agreement with this finding, inflammation and insulin resistance might serve as mediators in the connection between SHBG and CKD according to the results of a recent Mendelian randomization study [30]. In addition, based on the findings of another Mendelian randomization study in UK Biobank data, genetically predicted higher levels of SHBG were linked to reduced CKD risk and improved kidney function in men, but not in women [31]. This indicates a potential gender-specific role of SHBG in CKD. It has also been found that testosterone levels had a positive correlation with insulin resistance in men with Type 1 diabetes [32].

Weak points of the study presented here are both its cross-sectional design and the relative low sample size, a significant limitation which may obscure other contributing factors, such as the duration of dialysis. In this context, the post hoc power analysis gave a power of 80% for non-parametric comparisons and for correlation analyses.

## Conclusions

Our findings confirm enhanced prevalence of biochemical hypogonadism in males on chronic hemodialysis. In non-diabetic cases the serum levels of SHBG correlated with serum insulin and insulin resistance. However, larger studies are needed to gain a deeper understanding of how androgens and SHBG might serve as modifiable risk factors for kidney function and CKD. Understanding the factors influencing SHBG levels and the underlying mechanisms could offer valuable insights into preventing and treating cardiovascular complications and CKD itself.

## Data Availability

No datasets were generated or analysed during the current study.

## Abbreviations

BMI: Body Mass Index
BUN: Blood urea nitrogen
CKD: Chronic kidney disease
eGFR: Estimated glomerular filtration rate
FAI: Free androgen index
HOMA-R: Homeostasis model assessment index
IRMA: Immunoradiometric assay
PCR: Protein catabolic rate
SHBG: Sex hormone binding globulin
UF: Ultrafiltration volume
WHO: World Health Organisation
